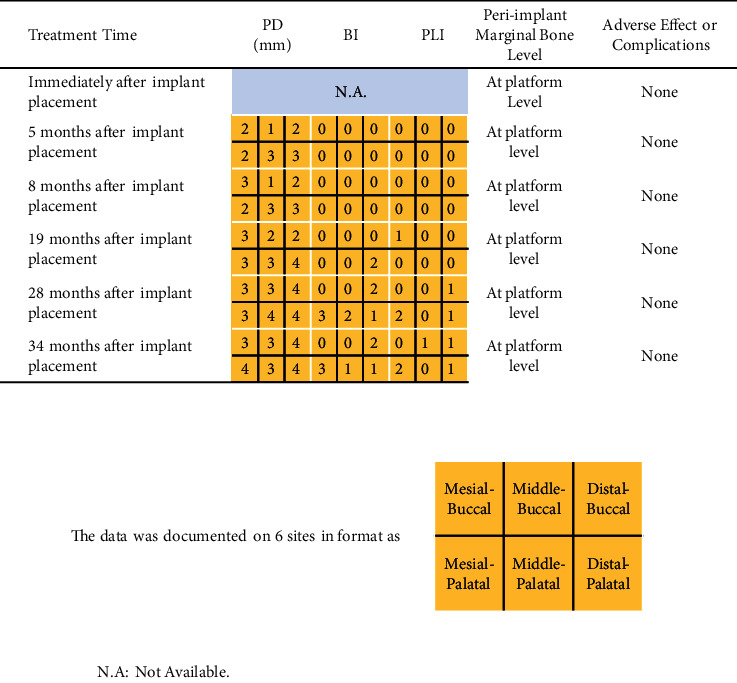# Clinical, Histological, and Radiological Outcomes of Sinus Floor Elevation Using a New Approach before Extraction of Periodontally Hopeless Maxillary Molars: A Case Report with 3-year Follow-Up

**DOI:** 10.1155/2022/8143765

**Published:** 2022-11-15

**Authors:** Zhaoguo Yue, Qi Liu, Haidong Zhang, Jingwen Yang, Lili Miao, Jianxia Hou

**Affiliations:** ^1^Department of Periodontology, Peking University School and Hospital of Stomatology, National Engineering Laboratory for Digital and Material Technology of Stomatology, Research Center of Engineering and Technology for Digital Dentistry of Ministry of Health, Beijing Key Laboratory of Digital Stomatology, Beijing, China; ^2^BYBO Dental Hospital, Beijing, China; ^3^Department of Prosthodontics, Peking University School and Hospital of Stomatology & National Clinical Research Center for Oral Diseases & National Engineering Laboratory for Digital and Material Technology of Stomatology & Beijing Key Laboratory of Digital Stomatology, Beijing, China

## Abstract

*Introduction*. This report is the first to present a case with 3-year follow-up, in which sinus floor elevation was performed before extraction of periodontally hopeless teeth, in order to shorten the edentulous interval between extraction and loading of implants and improve the patient's quality of life. *Case Presentation*. After a series of initial and supportive periodontal therapies, the lateral window was created at the apices of the hopeless teeth, followed by grafting of bone substitute and membrane material at the sinus floor. The tooth was preserved for 13 months prior to extraction followed by immediate implant placement. The patient is satisfied with the oral function partially retained during the treatment period. Predominance of new bone was detected by histologic analysis. The available bone height was augmented from 1–2 to 12–14 mm with little resorption (less than 2 mm of height) after 3 years of follow-up. The dental implant is in good condition without obvious signs of peri-implantitis or mobility after 3 years of loading. *Discussion*. The feasibility of modified sinus floor elevation (MSFE) could be seen in the current case. The potential benefit of MSFE may include shortening the edentulous interval, facilitating bone regeneration, and providing a chance for immediate implant placement. However, further clinical case evaluations and controlled studies are required to determine indications, effectiveness, and safety of such augmentation procedures.

## 1. Introduction

Severe alveolar bone loss has regularly been found around periodontally hopeless teeth, resulting in inadequate bone volume for implant placement after extraction [1–3]. For the posterior maxilla, when the residual bone height is less than 5 mm, it is typically necessary to perform lateral sinus floor elevation (LSFE) after extraction of compromised teeth [4–6]. A traditional two-stage LSFE may need 6–9 months for socket healing and osteogenesis at the sinus floor before placement of implants becomes possible [7, 8]. Concerning patients with advanced periodontitis whose molars are rated as hopeless, possibly so even bilaterally, long edentulous intervals between extraction and loading of implants may make them unwilling to accept dental implants. Especially when the compromised teeth are free of pain or abscess after initial periodontal therapy (IPT), residual function is critical, though limited, for the patient's quality of life [9, 10]. As a result, patients, as well as dentists, may hesitate regarding the timing of extraction.

This report presents a new modified technique of LSFE (modified sinus floor elevation [MSFE]), which is performed before the extraction of compromised teeth. This allows clinicians to delay the extraction and shorten the edentulous interval, making LSFE more acceptable for patient.

## 2. Clinical Presentation

**Figure 1:**
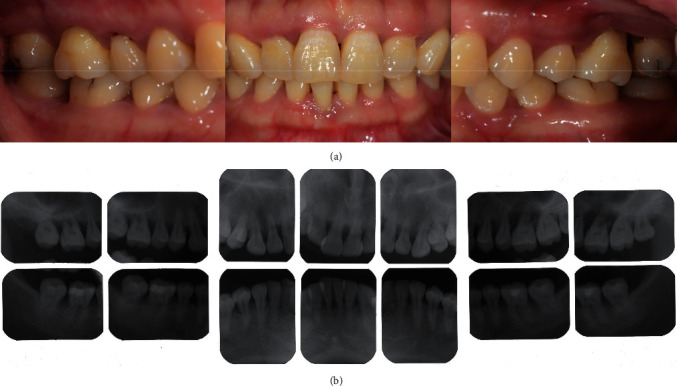
The patient had been diagnosed with aggressive periodontitis 4 years ago. Teeth #16 and #26 were severely compromised with bone loss to the apical third of root. (a) Intraoral photographs. (b) Periapical radiographs.

**Figure 2:**
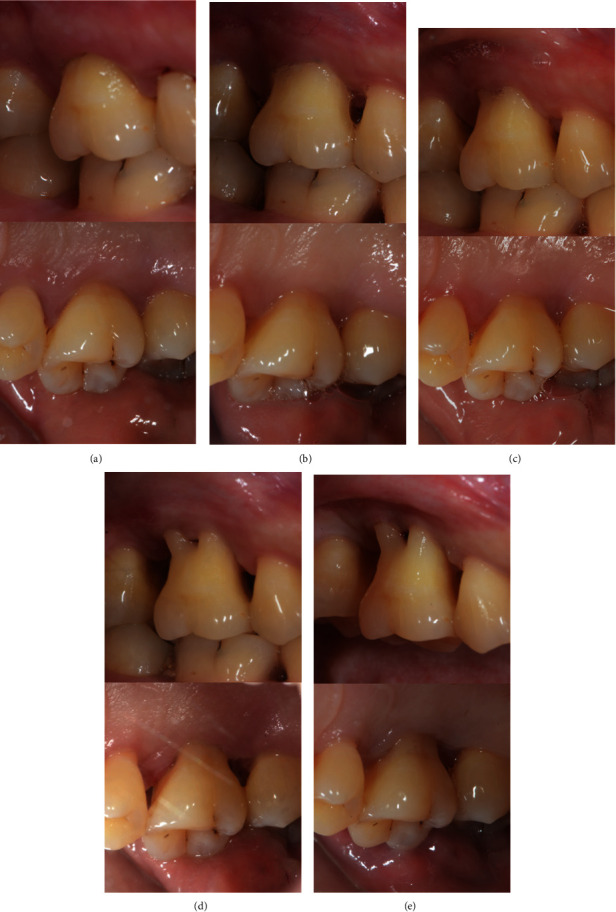
Tooth #16 acquired periodontally healthy status after active periodontal treatment. (a) Before periodontal treatment. (b) After 3 months of initial periodontal treatment (IPT) and supportive periodontal treatment (SPT). (c) After 5 months of IPT and SPT. (d) After 7 months of IPT and SPT. (e) After 10 months of IPT and SPT, a remittent status was acquired before the surgery.

A 33-year-old female presented to the Department of Periodontics of Peking University School of Stomatology, Beijing, China, on June 15, 2017, complaining chiefly about loose molars. Her teeth #16 and #26 had class II–III mobility, and probing depth around the teeth reached 6–10 mm. The alveolar bone of teeth #16 and #26 was resorbed to the apical third of root so that both teeth were rated as hopeless (Figure 1) [11]. Although the periodontal status was compromised, pulp testing revealed a normal response to thermal stimulation. Since teeth #16 and #26 were free from other discomfort and the chewing function was partially preserved, the patient was unwilling to have the molars extracted bilaterally. She was systemically healthy and a nonsmoker with no history of rhinitis or sinusitis. Her treatment consisted of IPT and frequent supportive periodontal therapy (SPT). We preserved tooth #16 until the restorative treatment period. Thorough descaling and root planing were performed on tooth #16. Follow-up visits between short intervals to control biofilm accumulation were scheduled every month. After the gingiva turned pink and firm without bleeding upon probing or deepening pocket (Figure 2), the periodontal status was improved to a remittent level [12], and MSFE was performed on tooth #16. Tooth #26 was extracted and restored by means of a conventional arrangement for LSFE and implant placement.

### 2.1. Modified LSFE

**Figure 3:**
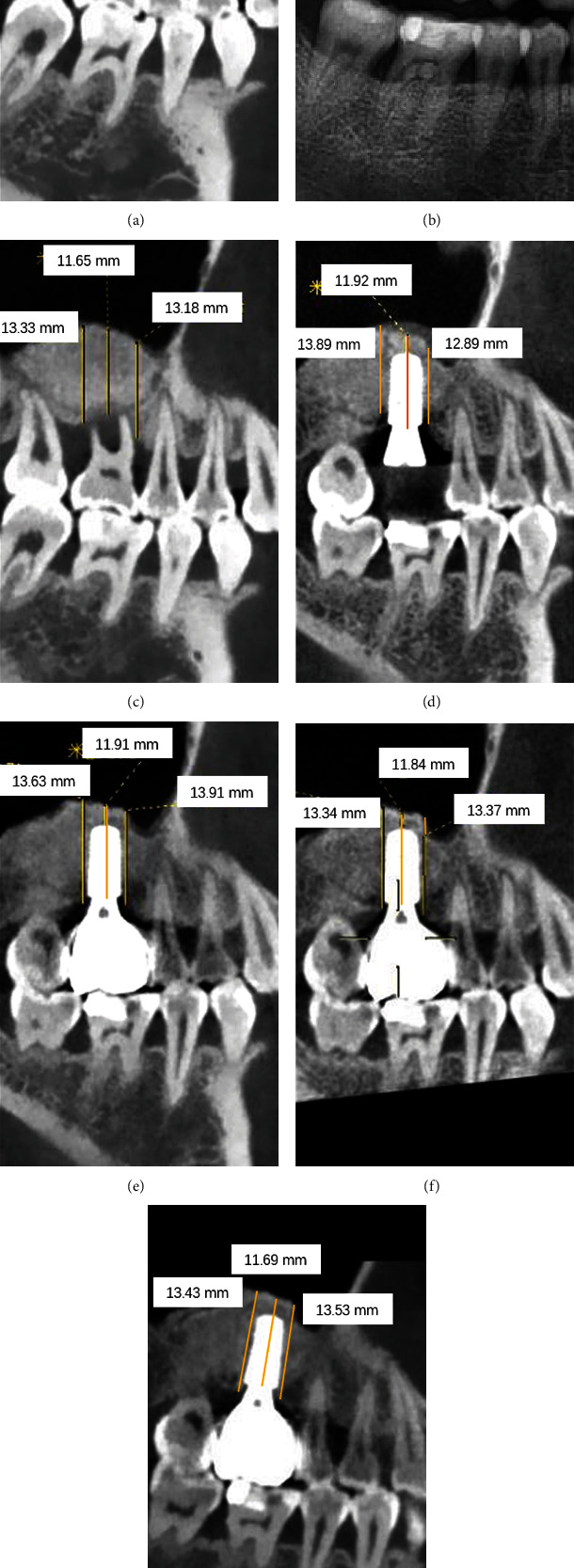
CBCT and pantomograph follow-up for tooth #16. (a) Before MSFE. (b) Immediately after MSFE (pantomograph). (c) 3 months after MSFE. (d) 10 months after MSFE. (e) 15 months after MSFE. (f) 26 months after MSFE. (g) 35 months after MSFE.

Preoperative cone beam computed tomography (CBCT) images were obtained for initial screening and evaluation of the residual bone height between sinus floor and alveolar crest near tooth #16, as well as the morphology of the sinus floor. The right maxillary sinus was found clear and healthy with intact sinus floors. Only 1–2 mm of available bone height was observed, indicating LSFE before implant placement (Figure 3(a)).

**Figure 4:**
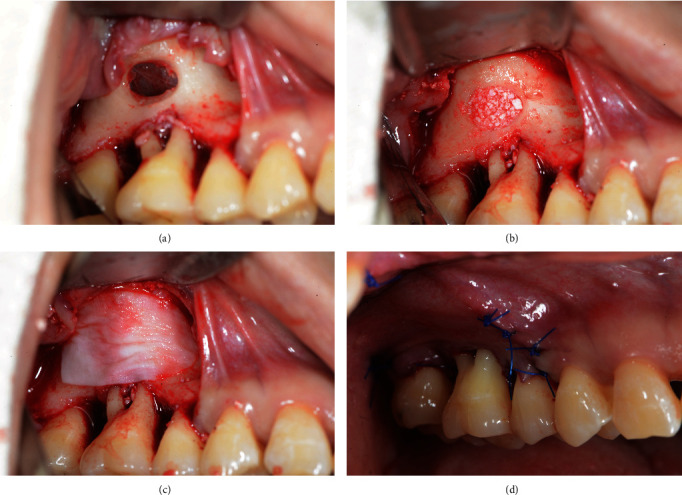
The process of the modified lateral sinus floor elevation. (a) Access window on the lateral wall. (b) Grafting materials at the sinus floor. (c) Collagen membrane covering access window. (d) Suturing.

The MSFE procedure on tooth #16 was performed based on Tatum's method [13]. Under local anesthesia, a crevicular incision was performed, which was designed to be extended to at least one adjacent tooth both mesially and distally. A full-thickness access flap was prepared, debridement of tooth #16 was performed, and an access window (5 mm × 8 mm) was created on the lateral wall of the maxillary sinus using a round diamond bur under irrigation with sterile saline. The lower edge of the window was formed at least 3–5 mm above the floor so as to keep it away from the teeth. The sinus membrane was elevated by at least 10 mm with curettes (Urban Sinus Lift Instrument, Hu-Friedy Mfg. Co., Chicago, IL, USA) from various angles. The elevation range exceeded the apex of the tooth (both buccolingually and mesiodistally). Then, the sinus membrane was lined with a layer of resorbable collagen membrane (Bio-Gide; Geistlich Pharma AG, Wolhusen, Switzerland) 13 mm wide and 25 mm long, and the generated cavity within the maxillary sinus was filled with 1.25 g of small particles of deproteinized bovine bone mineral (Bio-Oss; Geistlich Pharma AG). After grafting, another piece of collagen membrane 12 mm wide and 20 mm long was placed to cover the antrostomy defect. Interrupted sutures were created using non-absorbable suture material (PROLENE 4-0, Ethicon, Somerville, NJ, USA; Figure 4). The patient received antibiotic prophylaxis consisting of 500 mg amoxicillin (Amoxil Capsules, The United Laboratories Co., Hong Kong, China) 30 minutes before the surgery and 3 times daily for the following 7 days postoperatively.

**Figure 5:**
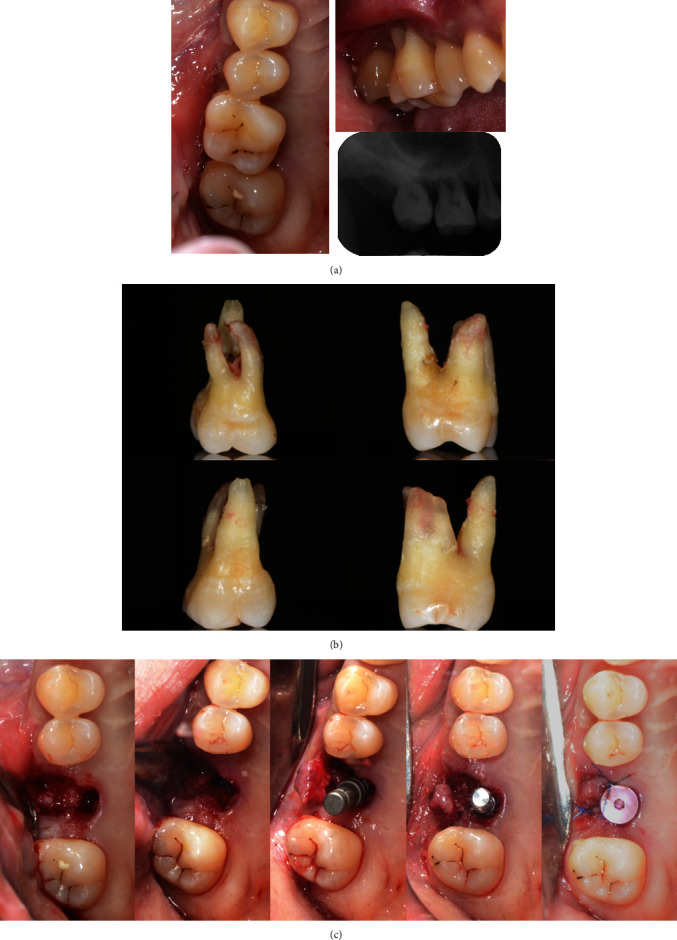
The process of implant placement. (a) Before the extraction. (b) The extracted teeth. (c) Immediate placement of implant.

After surgery, the status of tooth #16 was closely monitored. The patient was told to report any adverse event immediately upon occurrence and scheduled to receive SPT every 3 months. At each appointment, periodontal status including PD, BI, and PLI was recorded, and pulp vitality was examined. Thorough descaling and root planing were performed to suppress biofilm formation, and hygiene instructions were given in order to enhance the patient's own plaque control. Although the patient missed the second appointment due to COVID-19, she still had her teeth descaled regularly at another clinic. After 10 months, the patient was summoned for the last evaluation of tooth #16 before implant placement. The disease remission status remained stable without sign of active progression, and the vitality was kept normal. The patient described neither discomfort nor complications during the interval, and reported partial chewing function remaining on the right side. A CBCT scan was taken, and an increased bone height of 12–14 mm could be observed at tooth #16. Thus, the site was rated ready for a dental implant (Table 1 and Figure 5(a)).

### 2.2. Implant Placement and Biopsy Sample Collection

**Figure 6:**
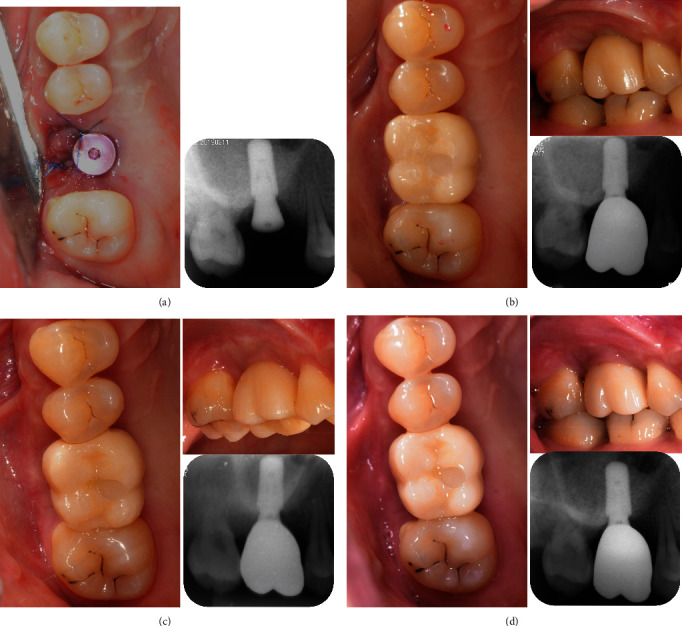
Periapical photographs follow-up for tooth #16. (a) Immediately after implant placement. (b) 5 months after implant placement. (c) 17 months after implant placement. (d) 26 months after implant placement.

First, tooth #16 was atraumatically extracted with solely vertical force to prevent pressure on the socket walls. After thorough scraping off of all the granulation tissue, the socket walls were confirmed to be intact. Then, a biopsy was taken with a hollow trephine with an internal diameter of 2 mm and a length of 6 mm, and the sample was liberally irrigated with sterile saline before it was fixated in phosphate-buffered formaldehyde. An implant hole was prepared simultaneously. A dental implant with a diameter of 4.8 mm, a length of 10 mm, and a coarsely sand-blasted, acid-etched surface was inserted in the planned site, with a depth where the platform reaches 1 mm below the buccal plate. The implant was inserted with a torque of 35 N·cm. Considering that the gap that remained between walls and implant was less than 2 mm, no bone substitute was filled into it [14]. Finally, the implant was covered with a healing abutment and sutured with non-absorbable sutures (PROLENE 4-0, Ethicon; Figure 5). To double-check the implant position, post-operation periapical radiographs using the paralleling technique were taken (Figure 6(a)).

**Figure 7:**
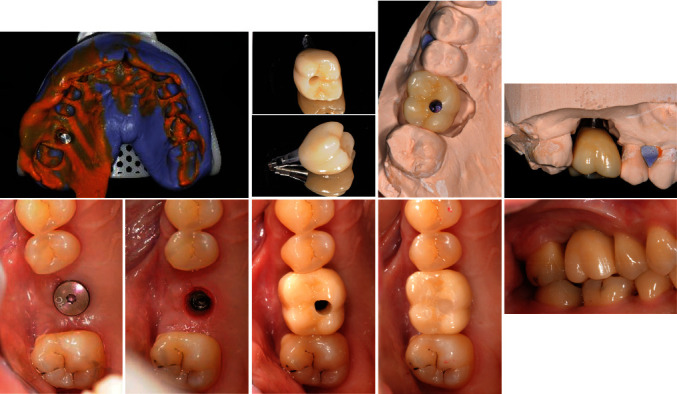
The process of delivering the superstructure.

After 4 months of implant healing without loading, the impressions and bite registration were taken with vinyl-polysiloxane material (Silagum & O-bite, DMG, Beijing, China). A cementable abutment (RC Cementable Abutment, Straumann Holding AG, Basel, Switzerland) with a height of 5.5 mm was selected for building the superstructure. Then, a working cast was scanned, and an anatomic zirconia crown was designed and fabricated using computer-aided design (DentalCAD, exocad GmbH, Darmstadt, Germany) and manufacturing system (Linggong, Fujian, China). A screw access was designed on the occlusal surface of the crown. After that, universal single-component priming agent (Monobond Plus, Ivoclar Vivadent, Schaan, Liechtenstein) was applied to the zirconia crown and titanium abutment accordingly. Resin luting cement (Relyx U200, 3M ESPE, St. Paul, MN, USA) was used to lute the two components extraorally according to the manufacturer's recommendations. The superstructure was fixed on the implant with torque of 35 N·cm one month after the impression-taking, and postoperative instructions including oral hygiene care were given to the patient (Figure 7).

#### 2.2.1. Conventional LSFE and Implant Placement

**Figure 8:**
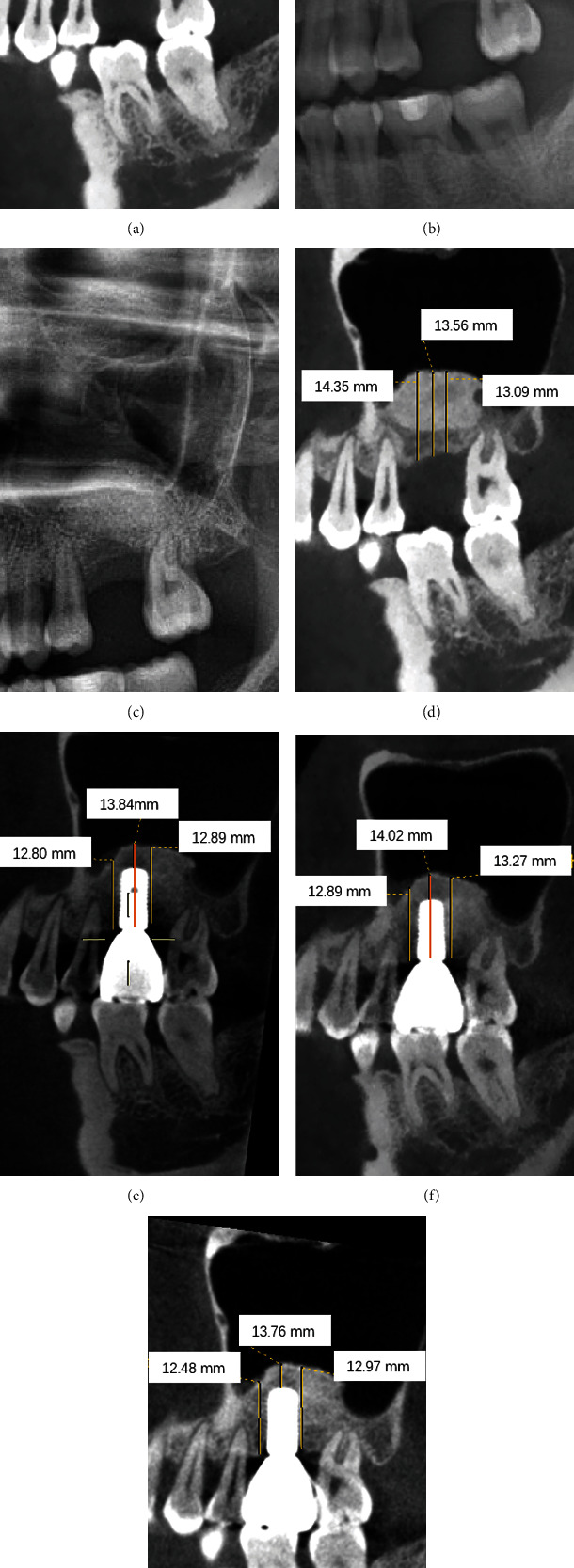
CBCT and pantomograph follow-up for tooth #26. (a) Before MSFE. (b) Immediately after MSFE (pantomograph). (c) 3 months after MSFE (pantomograph). (d) 7 months after MSFE. (e) 14 months after MSFE. (f) 20 months after MSFE. (g) 32 months after MSFE.

Conventional LSFE was carried out on tooth #26. The tooth was extracted in the initial therapy phase. Seven months after extraction, pre-surgical CBCT was taken. It revealed that the residual bone height remained about 1–2 mm (Figure 8(a)). Then, LSFE was performed, and the procedure was conducted by the same surgeon using the same technique and grafting materials as tooth #16. A layer of resorbable collagen membrane (Bio-Gide; Geistlich Pharma AG) with 13 mm wide and 25 mm long, together with 1.5 g of small particles of deproteinized bovine bone mineral (Bio-Oss; Geistlich Pharma AG), was applied at sinus floor.

After 6 months of healing, another CBCT was taken to observe the outcome of bone augmentation, which is nearly 12–14 mm high at implant site. Another dental implant with a diameter of 4.8 mm, a length of 10 mm, and a sand-blasted, large-grit, acid-etched surface (SLActive®, Straumann Holding AG) was placed at site with a depth where the platform was aligned with marginal bone. Meanwhile, a bone biopsy was taken as well. The implant was inserted with a torque of 35 N cm and submerged.

After 4 months of the unloaded implant healing, stage-two surgery was performed under local anesthesia to replace the implant cover screws with the healing abutments. After another month, impression was taken, and a crown was fabricated using the same workflow as tooth #16.

### 2.3. Clinical and Radiological Evaluation

Approximately 6 months after the MSFE/LSFE, the patient was called back for follow-up visits. A visual analog scale (VAS) evaluating oral function over the whole treatment period was handed over to the patient. The patient was asked to rate her oral function level using a 10-grade scale from “0—satisfying” to “10—discomfortable.” Clinical evaluation including mucosa conditions and complications, and radiological evaluation including periapical radiographs and CBCT (or pantomograph) were obtained at each follow-up visit.

### 2.4. Histological Evaluation

The bone biopsy samples were collected during dental implant surgery with the use of a hollow trephine, fixated in 10% formaldehyde (pH 7.4; +4°C), transferred to 70% ethanol, and stored until used for histomorphometric analysis. The samples were subsequently decalcified in 7% ethylenediaminetetraacetic acid, embedded in low-temperature-polymerizing methyl methacrylate, cut into sections (thickness 4 *μ*m), and stained with both hematoxylin–eosin and modified Mallory aniline blue. The histologic parameters were recorded as described in da Rosa et al. [15].

## 3. Outcomes

**Figure 9:**
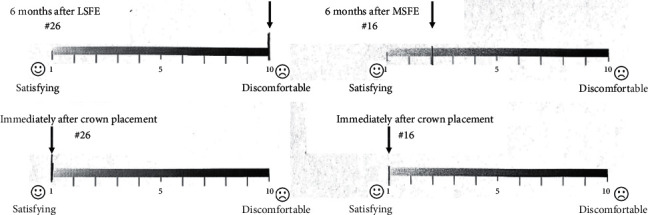
Visual analog scales (VAS) for discomfort were distributed to patient at 6 months after MSFE and immediately after crown placement.

**Figure 10:**
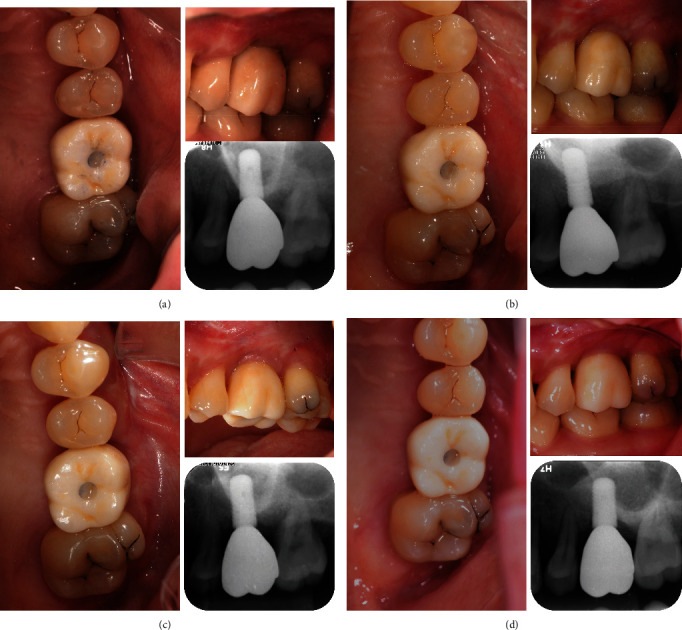
Periapical photographs follow-up for tooth #26. (a) Immediately after crown placement (5 months after implant placement). (b) 19 months after implant placement. (c) 28 months after implant placement. (d) 34 months after implant placement.

The patient is very satisfied with the whole treatment arrangement. No adverse event or complication was reported. At each re-visit before extraction of tooth #16, vitality of tooth #16 was detected as normal. The probing depth was limited within 3 mm with rare bleeding on probing, indicating a stable periodontal status without sign of progression (Table 1). The VAS was recorded as 2 at 6 months after MSFE, while the score was recorded as 10 on the opposite side (Figure 9). After nearly 3 years of loading, both implants are in good condition without obvious sign of peri-implantitis or mobility (Tables 1 and 2; Figures 6 and 10). The probing depth around teeth #16 and #26 ranged from 2 to 4 mm. The bleeding index occasionally reached 2–3 as plaque accumulating over time.

As for the radiological evaluation, adequate bone height was obtained at the sinus floor on both sides. Comparing with tooth #26, the same level of augmentation could be detected on tooth #16. The available bone height on both sides was augmented from 1–2 to 12–14 mm. Bone volume and marginal bone level after MSFE/LSFE remain stable during 3 years of follow-up. Little resorption (less than 2 mm of height) of bone substitute was observed (Figures 3 and 8).

**Figure 11:**
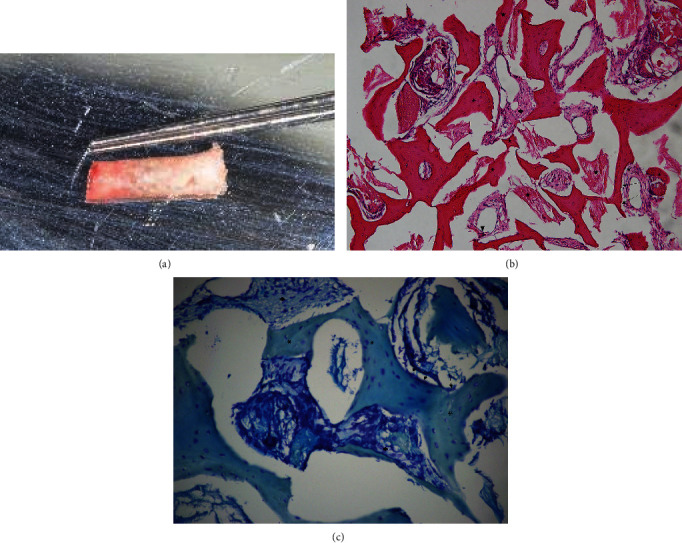
Histological evaluation of tooth #16. (a) The biopsy sample. (b) Representative section stained with hematoxylin-eosin (H&E) (×10). (c) Representative section stained with modified Mallory aniline blue (×20). Newly formed bone (asterisk), nonvital bone (star), blood marrow (diamond), bone lining cells (arrowhead), and an osteoclast (arrow) are visible.

Both sides shared a similarity in terms of histological analysis. The cylindrical biopsy sample collected from teeth #16 and #26 was found well-consolidated in macroscopic analysis. Newly formed bone as well as lines of osteoblast-forming osteoids could be observed on both sides at the apical side of the biopsy core. Lacunas and osteocytes could be seen in the area of immature bone, whereas limited nonvital bone with empty lacunae also remained in the view. On the MSFE side, scores were 3 for vital bone, 3 for mature newly formed bone, 2 for osteoblasts, 1 for osteoclasts, and 4 for bone marrow (Figure 11). On the LSFE side, scores were 4 for vital bone, 3 for mature newly formed bone, 1 for osteoblasts, 1 for osteoclasts, and 3 for bone marrow.

## 4. Discussion

Resorption of alveolar bone is a common consequence of periodontitis, which may leave behind inadequate bone volume for implant placement [16]. Especially for maxillary molars, poor periodontal status could even be regarded as a potential predictor for LSFE [16]. In this clinical case, residual bone height at tooth #16 remained only 1–2 mm before extraction, indicating LSFE was inevitable to place an implant. Thus, a new modified LSFE before the extraction was performed in advance. The possibility of MSFE was foreseen by Beitlitum et al., who demonstrated effectiveness and safety of extending sinus floor augmentation to adjacent teeth beyond the edentulous area [17]. The 3-year follow-up of our case confirmed the feasibility and effectiveness of MSFE. The implant at tooth #16 functioned well with no adverse event or complication ever reported. CBCT revealed adequate increasement on bone volume at the side of MSFE. Furthermore, bone substitute augmented at the sinus floor remained stable with little resorption (less than 2 mm of height).

According to the traditional method of LSFE, compromised teeth are supposed to be extracted 3–4 months before any access to the sinus floor. When it comes to bone heights of only 4 mm or less (≤4 mm), two-stage surgery with bone grafting was required, which means at least another 6 months should elapse before implant placement [7, 8]. This edentulous period in the posterior maxilla leads to obvious reduction of masticatory efficiency. More specifically, the absence of a second molar may cause 5–7% decrease of chewing efficiency [18], whereas a premolar–first molar region bears 90% of the masticatory function of the dentition [19]. This is vital for patients with advanced periodontitis, since bilateral molars are more likely to be affected than other teeth [1]. For the current case, the VAS score for oral function satisfaction was 2 on the right side as a result of the MSFE on tooth #16. The edentulous period was reduced to 5 months, versus 16 months on the left side which got a VAS score of 10. By arranging LSFE before extraction, limited but vital function of the molar tooth #16 was fully utilized, and oral function during the treatment intervals was improved.

MSFE may also offer the option of immediate implant placement. The bone volume augmented beforehand may be used to achieve initial implant stability, which is essential for successful immediate placement [20]. Fresh sockets after extraction could be practically suitable for inserting implants if the MSFE sites meet with basic conceptions for immediate placement: (a) adequate bone volume after MSFE for selection of proper implant length and width, as well as its initial stability; (b) absence of endodontic disease or progression of periodontal disease; (c) intact socket walls; and (d) thick biotype with more than 2 mm width of keratinized mucosa. In such case, advantages of immediate placement generally recognized by previous studies [21–24] could be exploited on the site, such as earlier loading of the implant or preservation of alveolar bone contour. In current case, another 3–4 months are saved compared with delayed placement.

Bone remodeling is supposed to start upon tooth extraction [25]. Without functional stimulus, the alveolar process continues to retrude towards the basal bone [2, 3]. MSFE minimizes the period of function deficiency and, therefore, has the potential to halt the remodeling at early stage. Moreover, the tooth extraction was shown to abet the pneumatization process of the maxillary sinus due to the absence of stimuli from biting forces [26, 27]. With the tooth kept on site after modified LSFE, biting forces continue to act, providing a chance for preventing resorption of regenerated bone or preventing “re-pneumatization.” Thus, quality and quantity of the bone augmented on sinus floor with a modified LSFE technique may be more promising than traditional methods are. In the current case, predominance of new bone was detected via histologic analysis on both sides, with lacunas, osteocytes, and lines of osteoblast-forming osteoids present. Similar histological findings and a comparable level of osteogenesis could be observed on MSFE side and LSFE side. Furthermore, controlled data and studies are necessary to prove or deny superiority of the histological manifestation over traditional approaches.

A potential adverse effect of MSFE may be pulp necrosis. It was reported that elevation of sinus membrane under the teeth would possibly disrupt the innervation and blood supply of individual branches of root apices [28]. However, a retrospect study revealed that most of the pulp devitalization occurred due to severe carious or periodontal lesions. Only 0.7% of probability of tooth devitalization was rated due to sinus floor augmentation [29]. The periapical tissue will not be damaged as long as the integrity of alveolar bone around the apexes of roots remains intact [17]. In the current case, the surgical invasions were carefully confined to the sinus floor without interfering the apex of tooth #16. As a result, the vitality of tooth #16 was detected as normal during the whole period. More clinical data are needed to prove the safety of MSFE.

According to Carranza et al., teeth whose attachment is inadequate to maintain health, comfort, and function are regarded as “hopeless” and should be extracted sooner or later [11]. Once the biofilm was optimally controlled by IPT and frequent SPT, some of the “hopeless” teeth could be freed of acute symptoms and regain part of chewing function [29]. This status refers to reduction of inflammation, such as improvement of PD and control of bleeding on probing, although optimal control of local contributing factors is not achieved. At this time, periodontal disease remission sets in [11], and it is the situation that we consider to be the indication for modified LSFE, which could be summarized as teeth with: (a) more than 50% attachment loss; (b) poor crown-to-root ratio; (c) poor root form; (d) class II or III furcation involvements; (e) more than class II mobility; and (f) difficulty to clean by the patient himself or herself, but are stably manageable under frequent periodontal supportive treatment. Other indications for MSFE refer to the traditional perspective of lateral approach [4–6, 16, 17], such as: (g) inadequate interfurcal bone height for future implant replacement (≤6 mm) together with probing depth exceeding 6 mm; (h) endodontically healthy/treated; (i) absence of sinus disease (e.g., active sinus infection, recurrent chronic sinusitis, or neoplasms of the sinus); and (j) systemically healthy. More cases and long-term follow-up are necessary to prove the validity of the preliminary indication.

## 5. Summary

**Table:** 

Why is this case new information?	The modified sinus floor elevation approach offers an effective method to shorten the edentulous interval between extraction and loading of implants and improve the patient's quality of life, especially for patients with hopeless molars on both sides.
What are the keys to successful management of this case?	The periodontal status of the compromised teeth needs to be managed to a remittent level by means of IPT and SPT pre-operatively. Specifically, a significant decrease in inflammation, stabilization of disease progression, and partial restoration of the chewing function should be achieved before surgery.
What are the primary limitations to success in this case?	The difficulty of flap elevation may increase since the hopeless or loose teeth still remain on site.
	In some cases, the sinus membrane may be thickened or exhibit morbid changes due to chronic inflammation among molars.

## Figures and Tables

**Figure 12:**
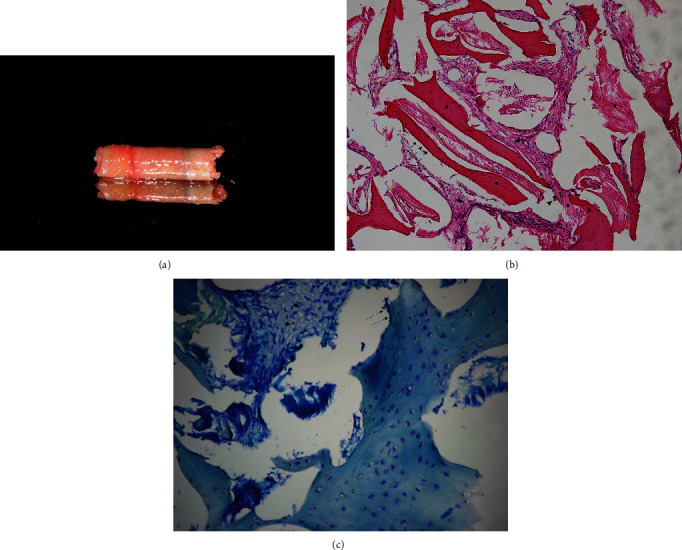
Histological evaluation of tooth #26. (a) The biopsy sample. (b) Representative section stained with hematoxylin-eosin (H&E) (×10). (c) Representative section stained with modified Mallory aniline blue (×20). Newly formed bone (asterisk), nonvital bone (star), blood marrow (diamond), bone lining cells (arrowhead), and an osteoclast (arrow) are visible.

**Table 1:** Clinical evaluation of tooth #16 at each follow-up visit.

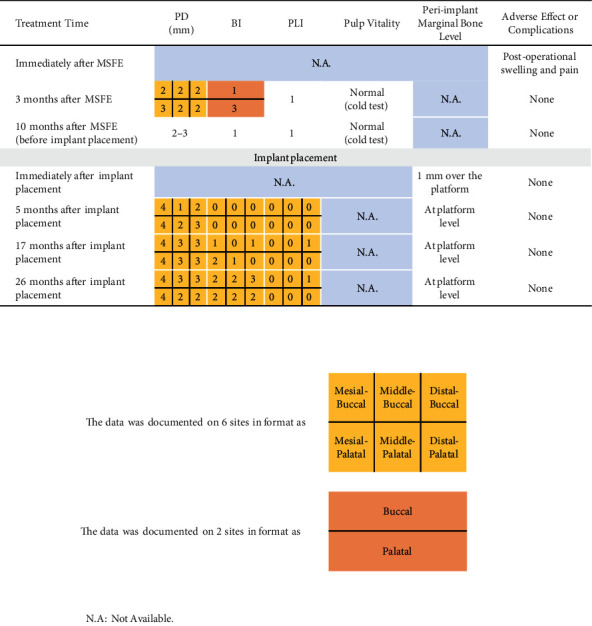

**Table 2:** Clinical evaluation of tooth #26 at each re-visit.